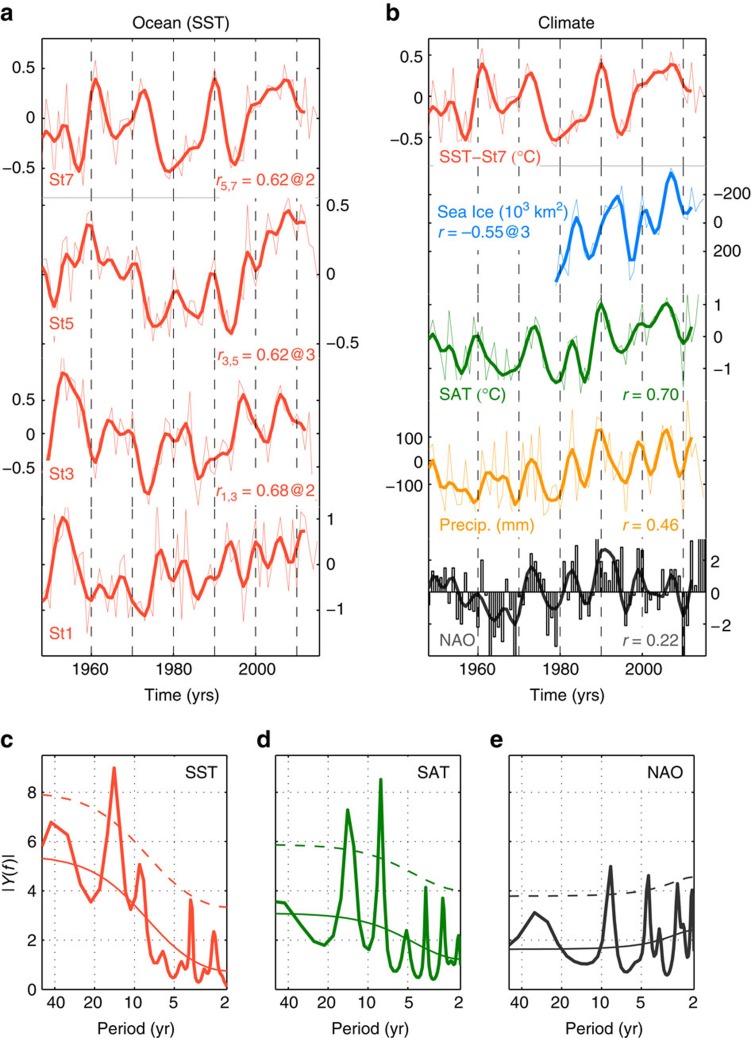# Skillful prediction of northern climate provided by the ocean

**DOI:** 10.1038/ncomms16152

**Published:** 2017-12-22

**Authors:** Marius Årthun, Tor Eldevik, Ellen Viste, Helge Drange, Tore Furevik, Helen L. Johnson, Noel S. Keenlyside

Nature Communications 8: Article number: 15875 ; DOI: 10.1038/ncomms15875 (2017); Published 06 20 2017; Updated 12 22 2017

In Fig. 2 of the original Article, information indicating the extent of the lagged correlations between low-passed and detrended time series was inadvertently omitted during the production process. The correct version of this figure appears below as Fig. 1.

## Figures and Tables

**Figure 1:**