# Supplementary material for: Skillful prediction of northern climate provided by the ocean
Source: Nat Commun. 2017 Jun 20;8:15875. doi: 10.1038/ncomms15875 (PMC5481837; doi:10.1038/ncomms15875)
Supplement: Supplementary Information [file ncomms15875-s1.pdf]

File name: Supplementary Information
Description: Supplementary Figures, Supplementary Tables and Supplementary References

File name: Peer Review File
Description:

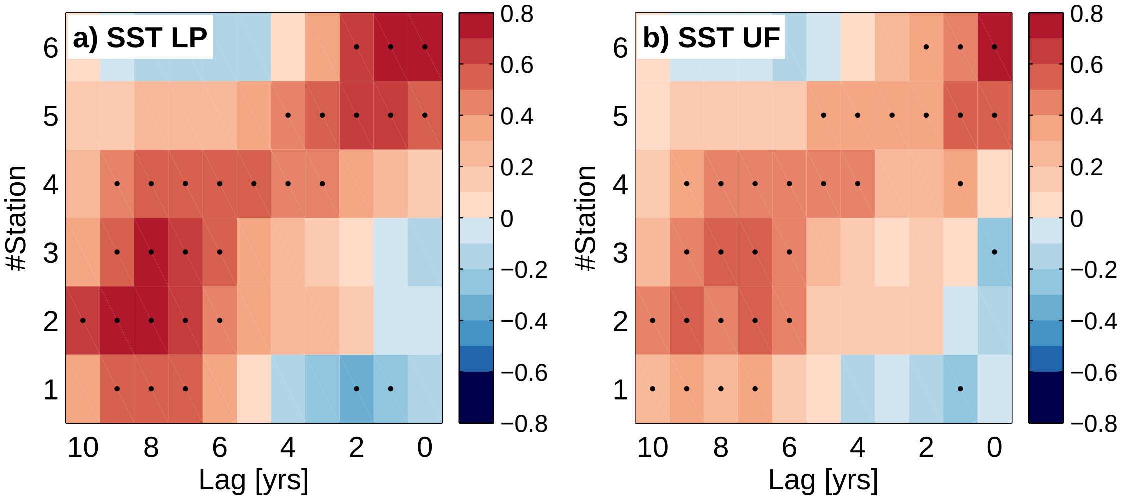

Supplementary Figure 1: a,b) Correlation between Norwegian Sea SST (St7; 1948–2012) and upstream stations in the subpolar North Atlantic (St1–6) for different lags. LP: 5-yr low-pass filter; UF: unfiltered. Black dots indicate significant correlations at the 90% confidence level.

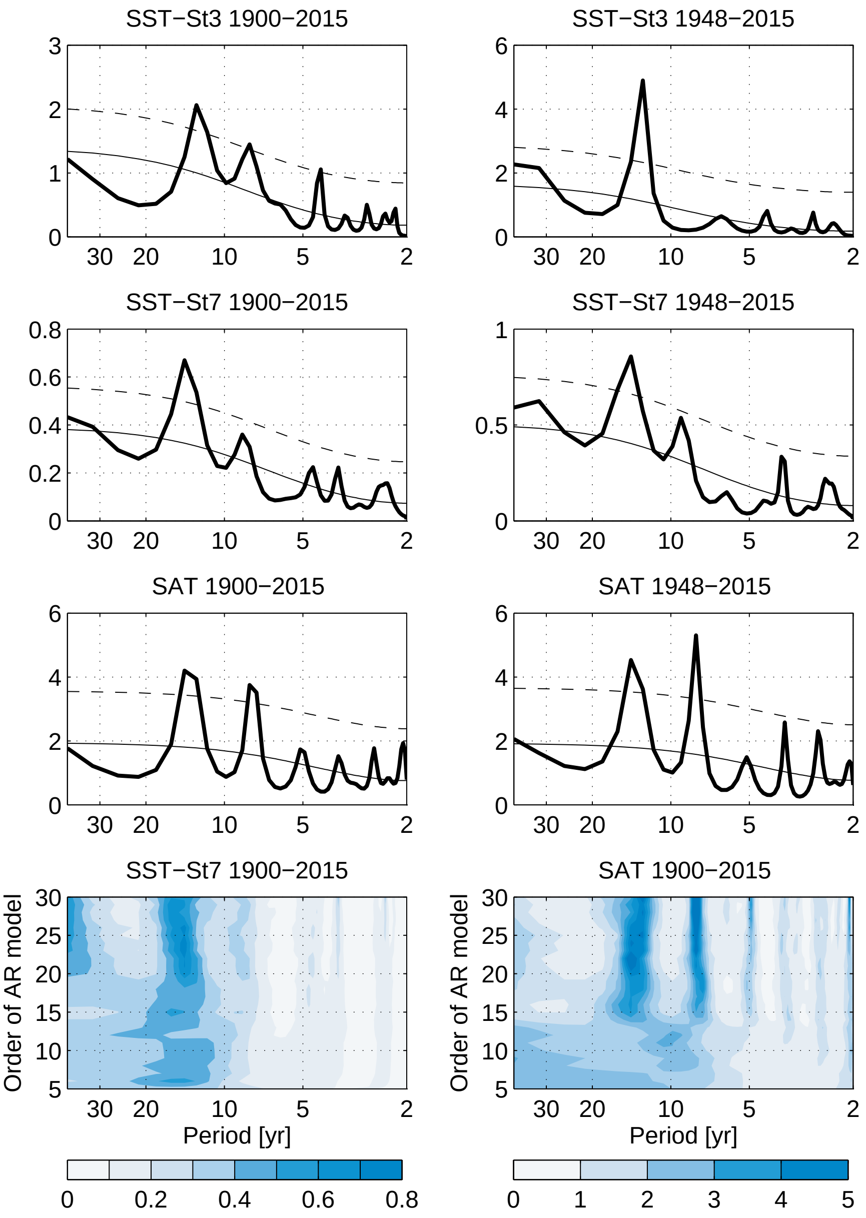

Supplementary Figure 2: Maximum entropy spectral analysis[1] for winter-spring SST in the subpolar North Atlantic (St3) and Nordic Seas (St7), and annual Norwegian SAT for different time periods. Each spectrum is compared with a theoretical red noise spectrum with a 95% confidence interval around the red noise (dashed line). The results of the spectral analysis, and especially the 14-year peak, are practically insensitive to the order of the autoregressive model[1] (lower panels; shading shows spectral power), implying that the spectral peaks are robust.

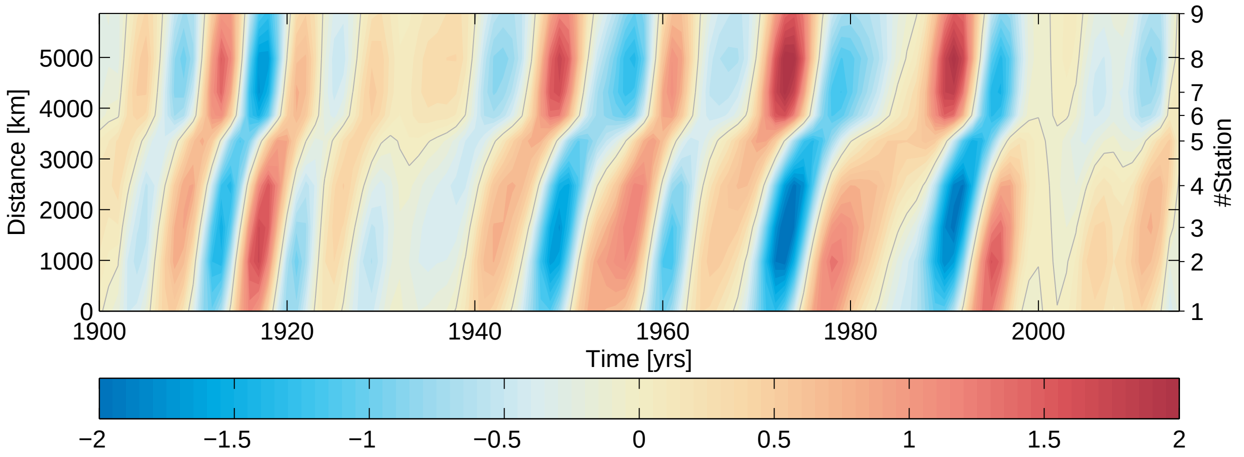

Supplementary Figure 3: Poleward propagation of temperature anomalies. Temporal development of the leading mode of SST propagation (first complex principal component) based on band-pass (5–40 years) filtered data between 1900–2012. The leading CPC mode explains 52% of the total (filtered) variance. The dominant time period is 14 years, and the mean northward propagation velocity is 3.5 cm s^{-1}.

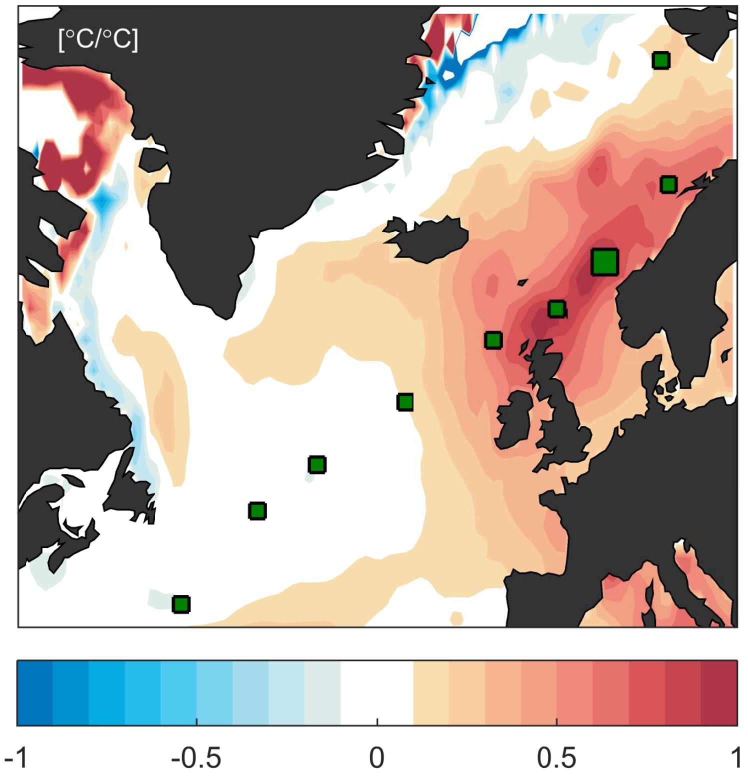

Supplementary Figure 4: Regressions of 5-year low-pass filtered winter-spring (December–May) SST onto the SST index at St7 (Norwegian Sea; larger green square) for the time period 1949–2012.

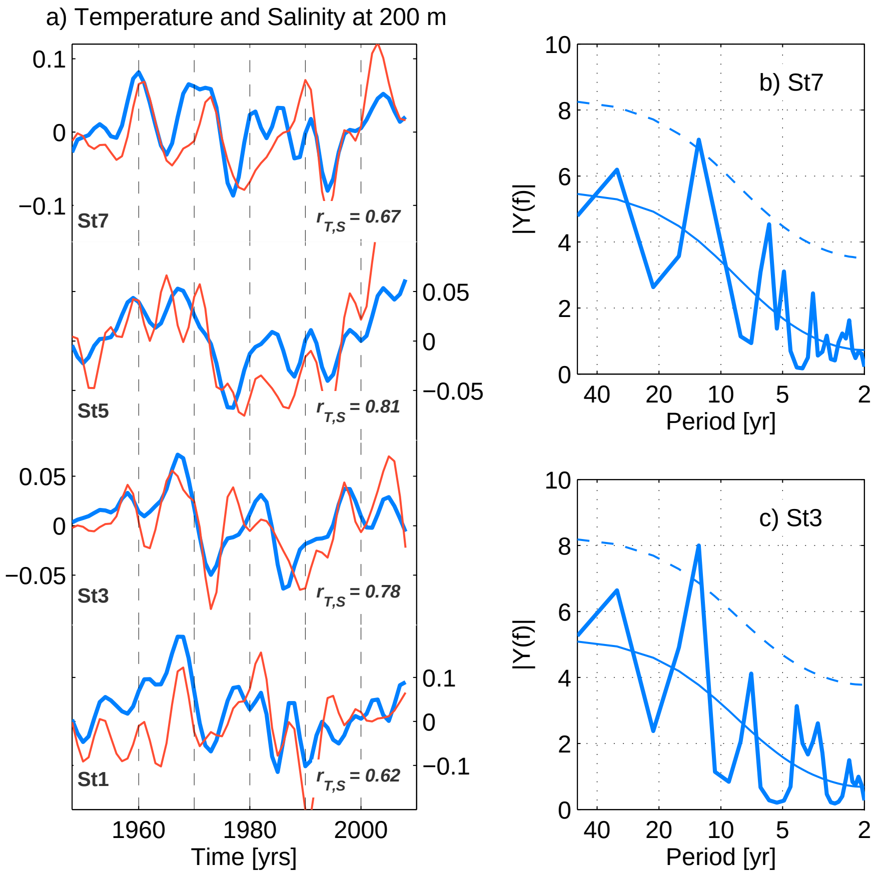

Supplementary Figure 5: a) Time series of sub-surface salinity (blue lines) and temperature (red lines) along the NAC-NwAC pathway from ref. 2. Temperature time series have been scaled for display purposes. Correlations between temperature and salinity are displayed. Time series have been 5-year low-pass filtered. Anomalies are relative to 1981–2010 climatology. We note that correlations are high between temperature time series from Ishii and HadISST datasets. b,c) Salinity power spectra, estimated by the maximum entropy method, for the subpolar North Atlantic (St3) and Nordic Seas (St7) based on unfiltered data. Thin solid lines are the theoretical red noise spectrum computed by fitting a first order autoregressive process with a 95% confidence interval (thin dashed lines) around the red noise.

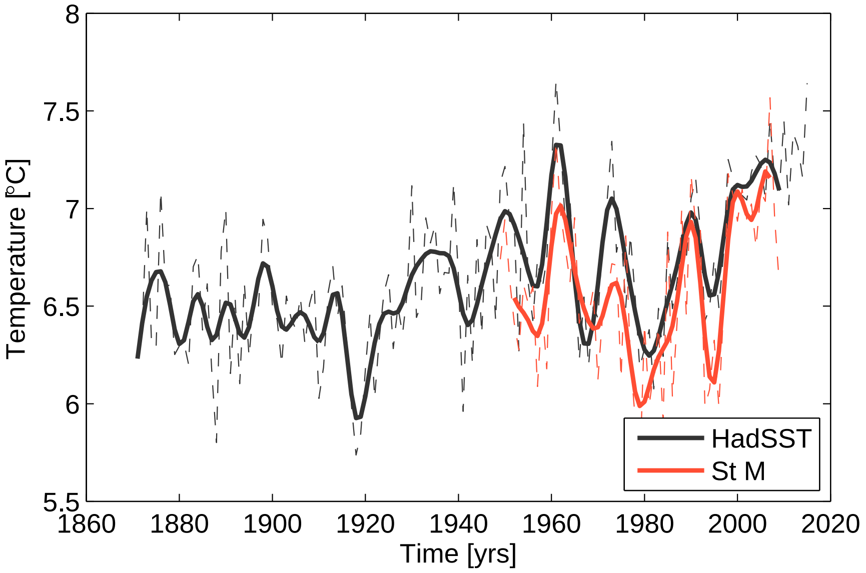

Supplementary Figure 6: Comparison between winter (December–February) HadISST (black) and observations from Station M (red; 66°N, 2°E) at 50 m. Thick lines are 5-year low-pass filtered. HadISST captures the observed interannual to decadal variability quite closely.

Supplementary Table 1: Geographic position of selected SST stations

Station	St1	St2	St3	St4	St5	St6	St7	St8	St9
Lat [°N]	41.5	47.5	50.5	54.5	58.5	60.5	63.5	68.5	76.5
Longitude [°E]	-49.5	-40.5	-32.5	-21.5	-10.5	-2.5	3.5	11.5	10.5

Supplementary Table 2: Correlations between SST and SAT

Period	1948–2012		1903–2012	
Station	St7	St8	St7	St8
r(LP)	0.70	0.73	0.54	0.57
r(UF)	0.49	0.49	0.44	0.45

Correlations between winter-spring (DJFMAM) SST in the Norwegian Sea (St7 and St8; see Fig. 1) and annual Norwegian SAT for different time periods. LP: 5-yr low-pass filter; UF: unfiltered. Listed correlations are significant at the 95% confidence level.

Supplementary Table 3: Correlations between SST and precipitation

Period	1948–2012		1903–2012	
Station	St7	St8	St7	St8
r(LP)	0.46	0.50	0.35	0.38
r(UF)	0.29	0.25	0.28	0.23

Correlations between winter-spring (DJFMAM) SST in the Norwegian Sea (St7 and St8; see Fig. 1) and annual Norwegian precipitation for different time periods. LP: 5-yr low-pass filter; UF: unfiltered. Listed correlations are significant at the 95% confidence level.

Supplementary References

1. Thomson, R. E. & Emery, W. J. *Data analysis methods in physical oceanography* (Elsevier, 2014).

2. Ishii, M., Kimoto, M., Sakamoto, K. & Iwasaki, S.-I. Steric sea level changes estimated from historical ocean subsurface temperature and salinity analyses. *J. Oceanogr.* **62**, 155–170 (2006).
